# Supplementary material for: Determination of the Pharmacokinetics and Pharmacodynamics of Isoniazid, Rifampicin, Pyrazinamide and Ethambutol in a Cross-Over Cynomolgus Macaque Model of Mycobacterium tuberculosis Infection
Source: Pharmaceutics. 2022 Nov 30;14(12):2666. doi: 10.3390/pharmaceutics14122666 (PMC9780811; doi:10.3390/pharmaceutics14122666)
Supplement: Supplementary file 1 [file pharmaceutics-14-02666-s001.zip › pharmaceutics-2043452-supplementary.pdf]

Supplementary data

Supplementary Table S1: PK parameters of TB drugs in Rhesus Macaques

Study	Treatment (s)	Drug, dose, dosage form	Blood Cmax (mean, ng/mL)	Tmax (mean, h)	$AUC_{0\text{-}inf}$ (calc, mg.h/L)	CL/F (L/h/kg) [%RSE]	V (L/h/kg) [%RSE]	KA (h^{-1}) [%RSE]	Terminal Half-life (h)
1	R 10	RIF, 10 mg/kg solution	1460	1.8	10.5	0.95 [8.4]	4.16 [19.4]	1.15 [39.5]	3.0
	Z 30	PZA 30 mg/kg solution	7803	1.4	27.0	1.11 [8.6]	1.35 [3.7]	0.82 [10.0]	0.8
	R 10, Z 30	RIF 10 mg/kg solution	2658	2.9	31.7	0.31 [154]	1.78 [234]	2.29 [646]	3.9
		PZA 30 mg/kg solution	7317	1.5	24.5	1.23 [8.0]	1.91 [16.7]	1.14 [32.6]	1.1
2	R 75	RIF 75 mg/kg dry powder	23267	3.2	Not Fitted				
	R 150	RIF 150 mg/kg dry powder	38700	5.5	Not Fitted				
	Z 400	PZA 400 mg/kg dry powder	239500	1.4	850.3	0.47 [7.9]	1.27 [13.0]	3.70 [74]	1.9
	Z 600	PZA 600 mg/kg dry powder	249500	1.4	2060.6	0.29 [32.0]	2.48 [28.7]	1.59 [71.0]	5.9
3	H 10, R 10, Z 250<br>H 10, R 30, Z 250	INH 10 mg/kg dry powder	656	1.9	7.1	1.41 [19.2]	19.0 [12.4]	2.81 [66.2]	9.4
	H 20, R 10, Z 250<br>H 20, R 30, Z 250	INH 20 mg/kg dry powder	4962	2.0	47.9	0.42 [10.9]	2.74 [15.5]	1.22 [46.9]	4.6
	H 10, R 10, Z 250<br>H 20, R 10, Z 250	RIF 10 mg/kg dry powder	2093	2.7	18.3	0.55 [10.1]	3.23 [13.8]	2.24 [64.4]	4.1
	H 10, R 30, Z 250<br>H 20, R 30, Z 250	RIF 30 mg/kg dry powder	25298	2.8	232.3	0.13 [11.2]	0.53 [17.0]	0.58 [35.1]	2.8
	H 10, R 10, Z 250<br>H 20, R 10, Z 250<br>H 10, R 30, Z 250<br>H 20, R 30, Z 250	PZA 250 mg/kg dry powder	168180	1.8	1078.9	0.23 [7.7]	0.87 [11.0]	2.23 [44.5]	2.6
4	E 25	ETH 25 mg/kg dry powder	811	4.1	38.0	0.66 [41.8]	22.4 [32.8]	0.54 [54.6]	23.6
	E 75	ETH 75 mg/kg dry powder	2945	3.9	47.1	1.59 [10.0]	22.0 [18.2]	1.42 [48.5]	9.6

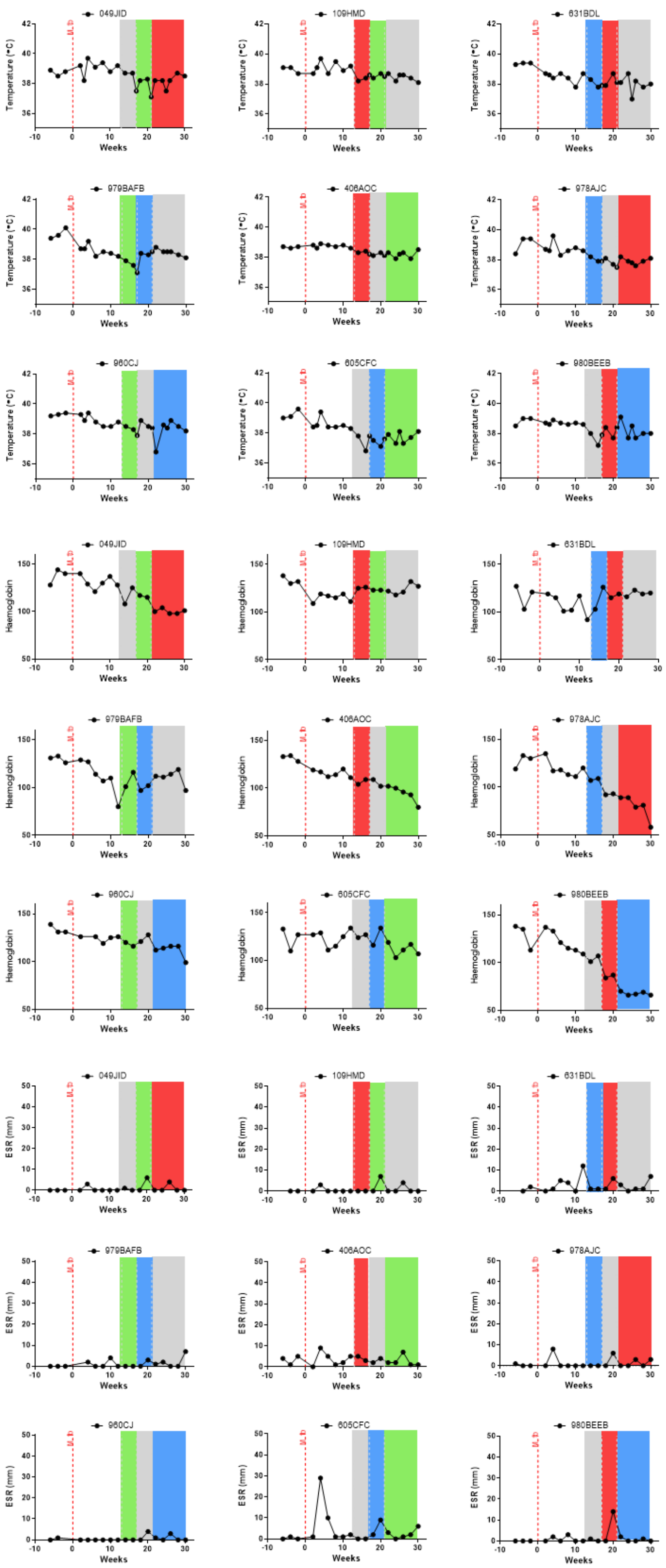

Supplementary Figure S1. ESR and Hb for Study 1.

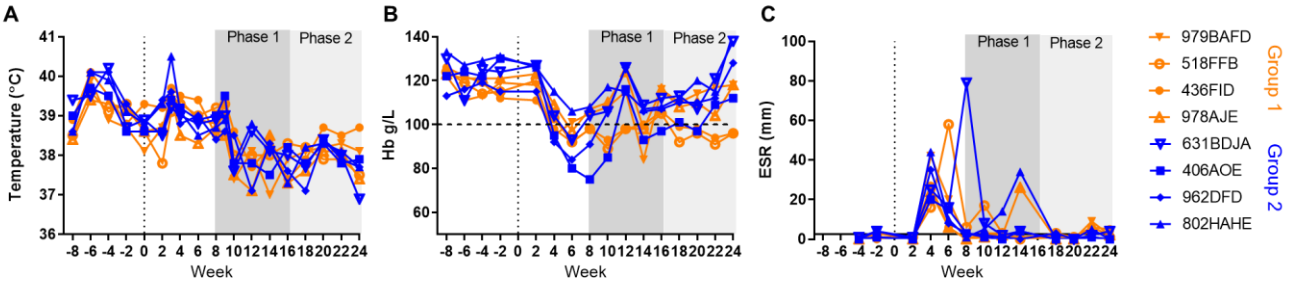

Supplementary Figure S2. Temperature, Hb and ESR for Study 2

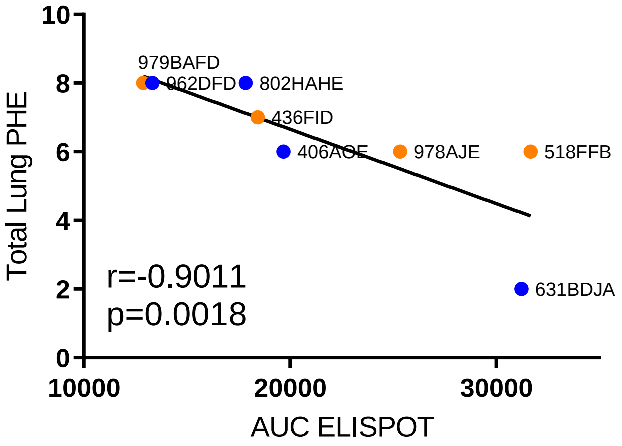

Supplementary Figure S3 – Study 2 ELISPOT correlations with lung pathology score.
